# Neuropsychological Responses in COA’s

**Published:** 1997

**Authors:** Sara Jo Nixon, Laura J. Tivis

**Affiliations:** Sara Jo Nixon, Ph.D., is director of the Cognitive Studies Laboratory, Oklahoma Center for Alcohol and Drug-Related Studies, Department of Psychiatry and Behavioral Sciences, University of Oklahoma Health Sciences Center, Oklahoma City, Oklahoma. Laura J. Tivis, Ph.D., is an adjunct faculty member in the Department of Psychiatry and Behavioral Sciences, University of Oklahoma Health Sciences Center, and a postdoctoral fellow in the Biological Psychology Graduate Training Program

**Keywords:** children of alcoholics, neuropsychological assessment, cognitive process, AODU (alcohol and other drug use) development, visual perception, spatial perception, learning, memory, problem solving, behavioral and mental disorder, family AODU history, alcohol use disorder classification, literature review

## Abstract

More than one-half of alcoholics may exhibit deficits in the processing of visual-spatial information, learning and memory, problem-solving and abstracting capabilities, and the regulation of goal-directed behavior. Research using nonalcoholic subjects suggests that some of these deficits may be familial and may predate the development of alcoholism. Results using alcoholic subjects are inconclusive, however, perhaps reflecting the effects of long-term alcohol consumption on cognitive performance. Factors that may influence the interpretation of results include psychiatric disorders, emotional instability, and type of alcoholism. Studies are needed to determine whether children from alcoholic families who become alcoholic themselves prove to be those who were previously most deficient in cognitive abilities.

The importance of genetic factors in alcoholism^1^ has prompted a search for markers for susceptibility to this disorder. Specific cognitive impairments revealed by neuropsychological testing may provide such markers.

Cognition refers to the processes involved in obtaining, organizing, and using information. These processes fall into various intellectual domains, such as verbal processing (e.g., learning lists or stories), visual-spatial processing (e.g., identifying similarities in pictures or figures), perceptual-motor skills (e.g., tracing maps or images), and abstracting or problem-solving (e.g., thinking about one’s actions or developing a plan for solving a problem).

Studies reveal that between 50 and 85 percent of detoxified alcoholics may exhibit deficits in one or more of these intellectual domains (Parsons 1987; National Institute on Alcohol Abuse and Alcoholism [NIAAA] 1989). It is unclear, however, whether these deficits in cognitive processing all result from the toxic effects of alcohol or whether some cognitive deficits precede the onset of drinking. Studies suggest that cognitive deficiencies similar to those observed in detoxified alcoholics may occur in some of their children before they use alcohol (Gillen and Hesselbrock 1992).

Although children of alcoholics (COA’s) are at increased risk for abusing alcohol, a significant percentage of them do not drink excessively (Tarter and Edwards 1986; Pihl and Bruce 1995). It is not known whether COA’s with preexisting cognitive problems similar to those of adult alcoholics are at greatest risk. Examining cognitive processes in COA’s is therefore important, because doing so may identify risk factors that predispose one to alcohol use, while revealing potential problems that COA’s might face in school. For example, COA’s with difficulties in problem-solving or emotional control may experience increased vulnerability to stress in social situations, leading to maladjustment and alcohol abuse (Wilson and Nagoshi 1988). In a detailed model of this possibility, Tarter and colleagues (1989) suggested that the interaction of family dynamics, peer influences, and intellectual functions may predispose adolescents to harmful alcohol use. Although research in this area continues, existing studies have contributed much to our understanding of the complexity of the problem. This overview highlights some relevant research; a complete review of the vast literature on this topic is beyond the scope of the article.

## Studies Using Nonalcoholic Subjects

Drejer and colleagues (1985) administered a battery of neuropsychological tests to 134 nonalcoholic sons of alcoholic fathers (i.e., family history positive [FHP] subjects) and to 70 nonalcoholic family history negative (FHN) subjects. Subjects were age 24 and older. Tests included measures of general intelligence, memory, attention, categorizing ability, and planning. Compared with FHN subjects, sons of alcoholics displayed impulsivity and a rigid, inflexible approach to problem-solving. The researchers concluded that nonalcoholics with a family history of alcoholism may have a diminished capacity for sustained goal-directed activity.

Studies of nonalcoholic COA’s also reveal impaired spatial perception similar to that found in alcoholics. In one study (Schandler et al. 1991), 17 FHP and 17 FHN subjects ages 25 to 42 were required to learn the positions of 8 nonsense shapes presented visually in different positions on a grid. Shapes were selected to minimize potential semantic associations, thereby emphasizing the visual-spatial aspect of the task. Family history was defined as alcohol abuse by both parents and at least one other relative. The FHP subjects performed significantly worse than FHN subjects on this task. In a similar study (Garland et al. 1993), FHP men required significantly more learning trials and made more errors than FHN men.

These patterns of deficits are similar to those previously reported among younger children of alcoholics. Schandler and colleagues (1988) found visual-spatial deficits among COA’s ages 6 to 11. Tarter and colleagues (1989) found that adolescent boys whose fathers were alcoholic performed more poorly on tests of visual scanning and attention, planning ability, and impulse control than did boys whose fathers were either clinically depressed or normal. More recently, Corral and colleagues (1996) found that children from families with a high density of alcoholism (i.e., an alcoholic father and two or more additional male alcoholic relatives) performed more poorly on tests of visual-spatial functioning and attention than did FHN children. Ozkaragoz and Noble (1995) reported poorer attention, visual-spatial, and memory performance among 10- to 14-year-old FHP males than among peer FHN controls. Peterson and colleagues (1992) found that college-aged men with, at minimum, an alcoholic father, paternal grandfather, and brother or uncle, performed more poorly than peer controls on tests that measured their ability to organize or classify new information.

Results of these studies suggest that cognitive deficits can predate chronic alcohol use. However, some studies have failed to find cognitive deficits in nonalcoholic COA’s (Gillen and Hesselbrock 1992; Schuckit et al. 1987; Workman-Daniels and Hesselbrock 1987).

## Studies Using Alcoholic Subjects

Despite the results of some earlier studies (Tarter et al. 1989; Alterman et al. 1986), most research has failed to confirm the hypothesis that the cognitive functioning of abstinent FHP alcoholics is worse than that of FHN alcoholics (Alterman et al. 1987; Reed et al. 1987). Differences in family history classification strategies may be one reason for the discrepancies found between some studies. Alterman and colleagues (1987) utilized four different family history classification strategies, representative of those frequently used by researchers, to assess neuropsychological functioning in chronic abstinent alcoholics. However, the researchers failed to find statistical differences between groups with any of their four family history classification models.

Using a large sample (i.e., 515 subjects), Schaeffer and colleagues (1988) initially found that FHP alcoholics were significantly more likely than FHN alcoholics to exhibit impaired abstracting ability. The researchers then randomly selected subjects from the original group to form 5 subgroups, each containing 40 FHP and 40 FHN subjects. Upon statistical reanalysis, only one of the five subgroups exhibited a significant association between FHP status and impaired abstracting ability. These results suggest that familial alcoholism effects are weak and inconsistent. Moreover, studies using an insufficient number of subjects are likely to miss whatever effects might exist.

Sinha and colleagues (1989) tested 143 male and female alcoholics using a battery of 16 tests to measure a range of cognitive abilities. The researchers found no significant correlation between neuropsychological performance scores and either personal or family drinking history. Similarly, Eckardt and colleagues (1995) detected no relation between familial alcoholism and cognitive ability in 101 alcoholic subjects.

In a later study, Drake and colleagues (1995) found no differences in cognitive ability between FHP and FHN alcoholics at admission to an alcohol treatment facility. Alcoholics were retested after 3 to 4 months of sobriety. Those FHP alcoholics who had resumed drinking performed significantly worse at followup than did their FHN peers. There were no differences in cognitive performance between FHP and FHN alcoholics who did not return to drinking. These data suggest that FHP alcoholics may be more vulnerable than FHN alcoholics to the effects of continued exposure to alcohol.

A recent study investigating cognitive efficiency among newly abstinent alcoholics and alcoholic polydrug abusers (Tivis et al. 1997) also failed to find differences between FHP and FHN groups. One aim of this study was to evaluate a cognitive *process* rather than traditional end-point neuropsychological measures. The former focuses on alcohol’s differential effects on underlying processes such as perceiving or retrieving information. Studies of end-point performance assess alcohol’s effects on certain skills associated with specific brain areas. Process approaches have been argued to be more sensitive than end-point measures to subtle deficits in cognitive function (Kaplan 1988).

As in the Alterman study mentioned previously (Alterman et al. 1987), Tivis and colleagues (1997) employed four different family history classification strategies. In the standard approach, subjects with at least one primary alcoholic relative or three secondary alcoholic relatives^2^ were considered to be FHP. In the lineality approach, subjects with alcoholism in a parent or grandparent on one side of the family were classified as unilineal and those with parental or grandparental alcoholism on both sides of the family as bilineal. In the multigenerational approach, subjects with no alcoholism in their family other than their own were classified as single; alcoholism in themselves and a parent, as double; and alcoholism in themselves, a parent, and a grandparent, as triple. Finally, in the quantitative method, subjects were assigned 1.00 point for each alcoholic primary relative and 0.50 point for each alcoholic secondary relative. The number of assigned points were divided by the total number of family members to obtain a numerical assessment of the subject’s family history status.

As a group, the alcoholics performed significantly less efficiently than a group of nonalcoholic control subjects of comparable age and education. However, the authors concluded that regardless of which classification system was used, family history did not account for the alcoholics’ efficiency deficits.

## Studies Comparing Alcoholic and Nonalcoholic Subjects

In an initial study, Schaeffer and colleagues (1984) compared 41 FHP and 27 FHN alcoholics with 19 FHP and 43 FHN nonalcoholic subjects on a battery of cognitive tests grouped into the following clusters: verbal, learning and memory, abstracting and problem-solving, and perceptual-motor. In general, alcoholics were significantly more impaired than nonalcoholic subjects, and FHP subjects performed more poorly than did FHN subjects. The FHN alcoholics and the FHP nonalcoholics were indistinguishable in their performance levels.

Yohman and Parsons (1987) investigated verbal reasoning abilities in FHP and FHN alcoholics and nonalcoholic control subjects. Overall, alcoholics demonstrated poorer verbal reasoning abilities than did the control group. Additionally, FHP alcoholics scored more poorly than either FHP control subjects or FHN alcoholics. In an attempt to cross-validate these findings, additional subjects were selected and given an identical test protocol. This time, the authors were unable to find the family history effects.

In one of the few studies of family history effects in women, Turner and Parsons (1988) administered verbal and nonverbal problem-solving tests to FHP and FHN female alcoholics and control subjects. Although the results did not reach statistical significance, a trend was clearly present: FHP alcoholics performed more poorly on both test types.

Taken together, these findings suggest that although some family history effects have been reported, they tend to be weak and difficult to replicate (Parsons et al. 1990; Tivis et al. 1993). Consequently, researchers have concluded that a family history of alcoholism is not reliably associated with impaired cognitive test performance.

## Interpreting the Results

Several confounding factors may affect the interpretation of neuropsychological test results. A possible factor, mentioned previously, is the diversity of methods used to classify subjects as FHP or FHN. As noted, however, recent data do not support this factor as a confounding variable (see article by Porjesz and Begleiter, pp. 236–240) (Tivis et al. 1997).

Possible confounding factors also include antisocial personality disorder (ASPD), childhood behavioral disorders (CBD’s), symptoms of depression and anxiety, and type of alcoholism. These factors are explored further in the remainder of this article.

### ASPD and CBD’s

Results of neuropsychological studies can be influenced by the presence of ASPD, a psychiatric disorder characterized by a pattern of irresponsible and antisocial behavior, and by CBD’s, a group of childhood psychiatric disorders characterized by any combination of the following: aggressiveness, hyperactivity, low self-esteem, and/or conduct problems.

ASPD is frequently associated with alcohol abuse and has been implicated in the development of certain types of alcoholism. Gillen and Hesselbrock (1992) investigated the effects of ASPD on neuropsychological functioning in both FHP and FHN young men. Results showed that a family history of alcoholism alone was not associated with neuropsychological impairment. Subjects with both familial alcoholism and ASPD, however, exhibited deficiencies in self-control and high-level verbal skills. The researchers noted that these deficiencies may impair a person’s ability to regulate behavior, which may contribute to a loss of control over alcohol consumption.

CBD’s have been associated with poorer cognitive performance, as well as a family history of alcoholism. For example, Glenn and Parsons (1989) observed that adult FHP alcoholics and nonalcoholics reported having more CBD symptoms than did a comparable FHN group. Researchers have also reported more CBD symptoms among alcoholics in general, compared with peer control subjects (Glenn et al. 1993; Glenn and Parsons 1989; Nixon et al. 1995). However, Nixon and colleagues (1995) found that symptoms of behavioral disorder could not account for cognitive impairment in alcoholics.

### Emotional Functioning

Symptoms of depression and anxiety are commonly associated with alcoholism. Because both of these emotional states affect cognitive functioning, their presence, either singly or combined, can influence the results of neuropsychological tests. For example, using a psychiatric diagnostic instrument called the Beck Depression Inventory, Sinha and colleagues (1989) confirmed that neuropsychological performance in sober alcoholics can be impaired by co-occurring depressive symptoms, especially in women.

Schafer et al. (1991) found that depressive symptoms were important predictors of cognitive performance, especially at the time of admission to treatment. Nixon and colleagues (1992) reported that alcoholics’ scores on an interpersonal problem-solving task were significantly and negatively related to depression scores (i.e., high depression scores were associated with low problem-solving test scores). Similarly, Tivis and Parsons (1995) reported a significant negative correlation between alcoholics’ scores on a measure of verbal-spatial functioning (as measured with a verbal test that requires spatial manipulations of objects) and a test of co-occurring anxiety. Thus, emotional dysfunction can have a substantial impact on neuropsychological functioning in alcoholics.

### Alcoholism Typology

For many years scientists have attempted to categorize types of alcoholism based on various distinguishing characteristics. For example, Cloninger and colleagues (1996) developed a two-part typology. Type I alcoholism is determined by both environmental and genetic influences, develops during adulthood, and affects both males and females. In contrast, type II alcoholism is predominately genetically determined and primarily affects sons of alcoholic men. It begins early, often during adolescence, and is usually associated with antisocial behavior. Type II alcoholics may exhibit the greatest deficits in neuropsychological studies. A complete review of this and other alcoholism typology systems is beyond the scope of this article.

## Summary

Alcoholism has been associated with deficits in the processing of visual-spatial information, learning and memory, problem-solving and abstracting capabilities, and the regulation of goal-directed behavior (Parsons 1987; Schandler et al. 1991). Research using nonalcoholic subjects suggests that these cognitive traits may be familial, predating the development of alcoholism. Results using alcoholic subjects, however, are inconclusive, perhaps reflecting the effects of long-term alcohol consumption on neuropsychological performance (Gillen and Hesselbrock 1992). Some factors that may influence the interpretation of results include ASPD, co-occurring emotional instability, and type of alcoholism. Studies that follow subjects over time are needed to determine whether FHP subjects who ultimately become alcoholic prove to be those who were previously most deficient in cognitive abilities.

## Footnotes

1 Multiple definitions exist for the terms “alcohol abuse” and “alcoholism”; therefore, the wording in each case is based on the terms used in the reference cited. See also the article by Porjesz and Begleiter, pp. 236–240.

2 Primary relatives include parents and siblings; secondary relatives include grandparents, uncles, and aunts.
